# Preoperative Diagnosis and Indocyanine Green (ICG) Fluorescence-Guided Surgery for Uterine Broad Ligament Hernia: A Case Report

**DOI:** 10.7759/cureus.80867

**Published:** 2025-03-20

**Authors:** Kosuke Kaneko, Michitoshi Takano, Tomomasa Fukasawa, Yoshin Koyama, Seiichi Yamagata

**Affiliations:** 1 Department of Surgery, Japan Community Healthcare Organization (JCHO) Tokyo Shinjuku Medical Center, Tokyo, JPN

**Keywords:** bowel, indocyanine green (icg), obstruction, surgery, uterus

## Abstract

Uterine broad ligament hernia is a rare cause of bowel obstruction in women, often challenging to diagnose preoperatively. We report a case of a 73-year-old woman presenting with abdominal pain and vomiting. CT imaging revealed an incarcerated small bowel loop within the Douglas pouch, with displacement of the uterus, leading to a diagnosis of strangulated bowel obstruction due to a broad ligament hernia.

An emergency laparotomy identified an ileal segment trapped in a defect of the left broad ligament. The bowel appeared ischemic, but intraoperative indocyanine green (ICG) fluorescence imaging confirmed adequate perfusion, allowing for preservation without resection. The hernial defect was repaired, and the patient recovered without complications.

This case highlights the importance of early CT diagnosis and the utility of ICG fluorescence imaging in assessing bowel viability, aiding surgical decision-making, and preventing unnecessary resection in cases of prolonged strangulation.

## Introduction

Uterine broad ligament hernia is a rare type of internal hernia, accounting for less than 4% of all cases [1]. It can lead to intestinal obstruction in women, posing diagnostic and therapeutic challenges. Preoperative diagnosis is often challenging, but CT can aid in its identification. Surgical management typically involves reducing the incarcerated bowel and closing the defect in the broad ligament. In cases of bowel ischemia or necrosis, resection may be necessary. Here, we report a case of strangulated bowel obstruction due to a broad ligament hernia that was diagnosed at an early stage, allowing for timely surgical intervention.

## Case presentation

Clinical presentation

**Figure 1:**
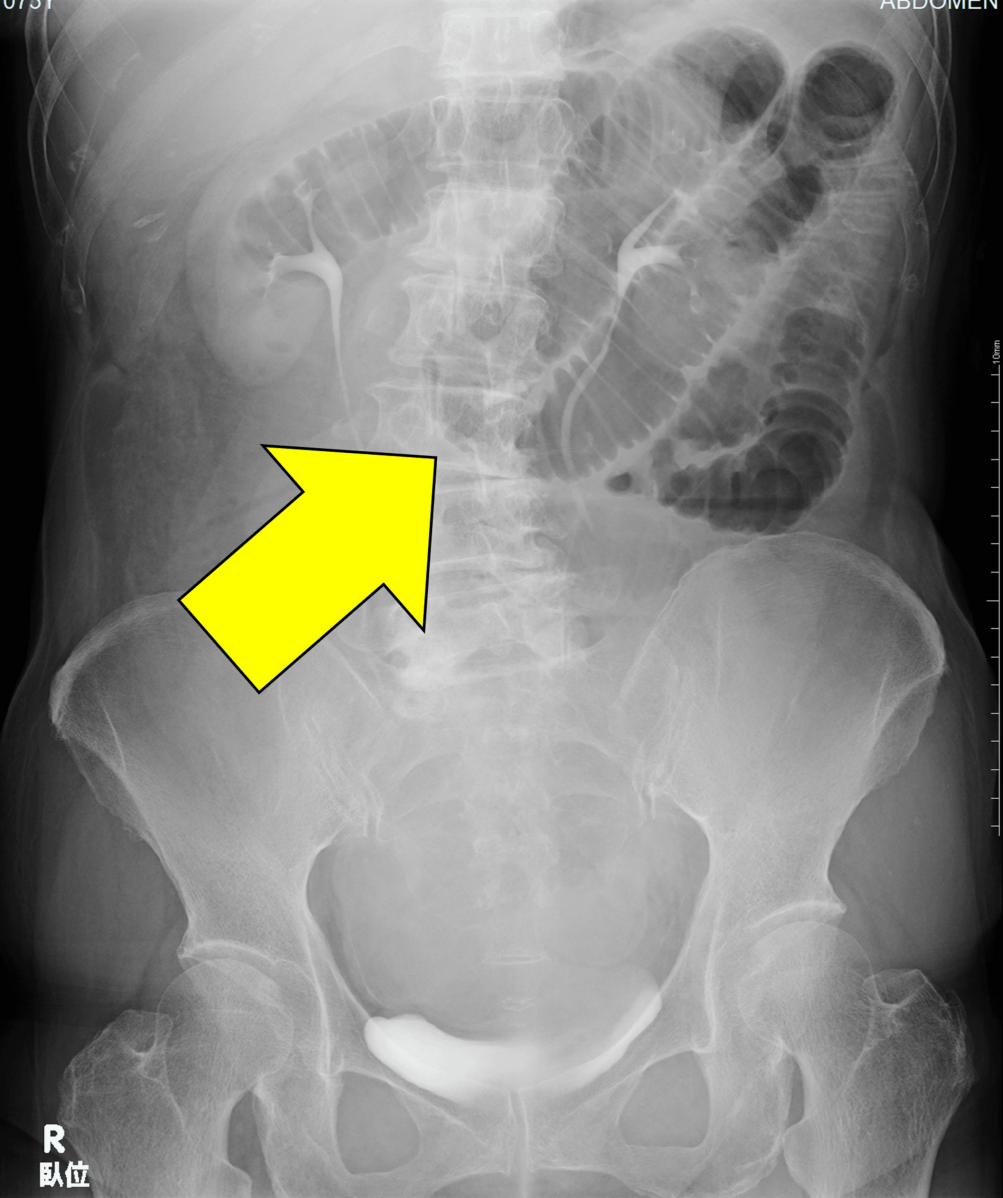
Abdominal plain X-ray revealed small bowel dilation (supine position).

A 73-year-old woman visited a local clinic with intermittent abdominal pain and vomiting. Initially diagnosed with acute enteritis, she received symptomatic treatment, but her symptoms persisted. The following day, she returned to the clinic, where an abdominal plain X-ray revealed small bowel dilation (Figure 1), raising suspicion of bowel obstruction. She was then referred to the emergency department of a secondary hospital, where she was diagnosed with strangulated bowel obstruction and transferred to our hospital for surgical intervention.

Clinical findings at admission

The patient was 160 cm tall and weighed 53 kg. Her blood pressure was 148/84 mmHg, pulse rate was 113 bpm, and body temperature was 36.8°C. Her abdomen was mildly distended with hyperactive bowel sounds. On physical examination, tenderness was observed across the entire lower abdomen, accompanied by guarding and rebound tenderness.

Laboratory findings

Blood tests showed a white blood cell count of 13,490/μL, a lactate level of 2.0 mmol/L, and a creatinine level of 0.96 mg/dL, indicating mild inflammation, elevated lactate levels, and renal impairment. No other significant abnormalities were detected (Table 1).

**Table 1:** Initial blood test results at the time of hospital admission. CRP: C-Reactive Protein; g/dL: Grams per deciliter; mEq/L: Milliequivalents per liter; BUN: Blood Urea Nitrogen; mg/dL: Milligrams per deciliter; eGFR: Estimated Glomerular Filtration Rate; mL/min/1.73m²: Milliliters per minute per 1.73 square meters; ALP: Alkaline Phosphatase; γ-GTP (GGT): Gamma-Glutamyl Transferase; AST: Aspartate Aminotransferase; ALT: Alanine Aminotransferase; LDH: Lactate Dehydrogenase; CK: Creatine Kinase; mmol/L: Millimoles per liter;  μL: Microliter; PLT: Platelet Count; NEUT: Neutrophils; LYMP: Lymphocytes; MONO: Monocytes; EOS: Eosinophils; BASO: Basophils; PT: Prothrombin Time; sec: seconds; INR: International Normalized Ratio; APTT: Activated Partial Thromboplastin Time; D-Dimer: D-Dimer Test.

Parameter	Unit and Value	Reference Range
Biochemistry		
CRP	3.425 mg/dL	<0.3 mg/dL
Total Protein	6.4 g/dL	6.5-8.0 g/dL
Albumin	3.2 g/dL	3.8-5.3 g/dL
Sodium	132 mEq/L	135-145 mEq/L
Potassium	3.6 mEq/L	3.5-5.0 mEq/L
Blood Urea Nitrogen (BUN)	25.7 mg/dL	8-20 mg/dL
Creatinine	0.96 mg/dL	0.6-1.2 mg/dL
eGFR (estimated)	59.7 mL/min/1.73m²	>60 mL/min/1.73m²
Total Bilirubin	0.6 mg/dL	0.2-1.2 mg/dL
ALP	103 U/L	38-113 U/L
γ-GTP	62 U/L	10-47 U/L
AST	43 U/L	13-33 U/L
ALT	13 U/L	8-42 U/L
LDH	227 U/L	119-229 U/L
CK	65 U/L	30-200 U/L
Lactate	2.0 mmol/L	0.5-2.2 mmol/L
Hematology		
WBC Count	2.99 ×10³/μL	3.5-9.0 ×10³/μL
RBC count	3.54 ×10⁶/μL	4.2-5.4 ×10⁶/μL
Hemoglobin	12.7 g/dL	12.0-16.0 g/dL
Hematocrit	38.0%	37-47%
Platelet Count (PLT)	56 ×10³/μL	150-400 ×10³/μL
Neutrophils (NEUT)	87.1%	40-70%
Lymphocytes (LYMP)	5.1%	20-45%
Monocytes (MONO)	5.7%	2-10%
Eosinophils (EOS)	0.5%	1-6%
Basophils (BASO)	0.3%	<1%
Coagulation		
PT (Seconds)	11.4 sec	10.0-14.0 sec
PT (%)	111.0%	70-130%
PT (INR)	0.94	0.85-1.15
APTT (Seconds)	35.4 sec	25-40 sec
D-Dimer	12.82 μg/mL	<1.0 μg/mL

Abdominal CT findings

**Figure 2:**
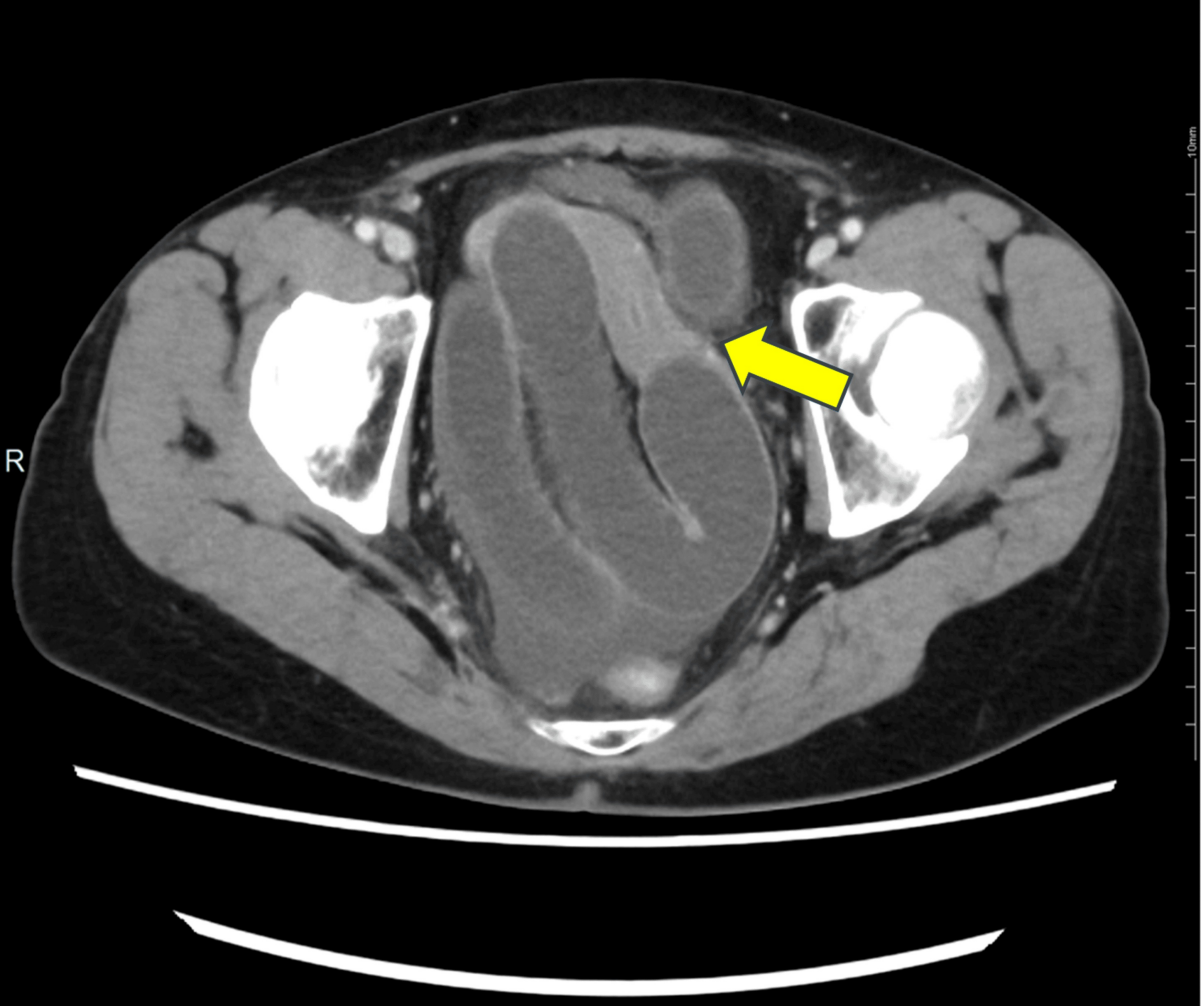
Contrast-enhanced abdominal CT revealed an ileal loop within the Douglas pouch, leading to anterior and rightward displacement of the uterus.

**Figure 3:**
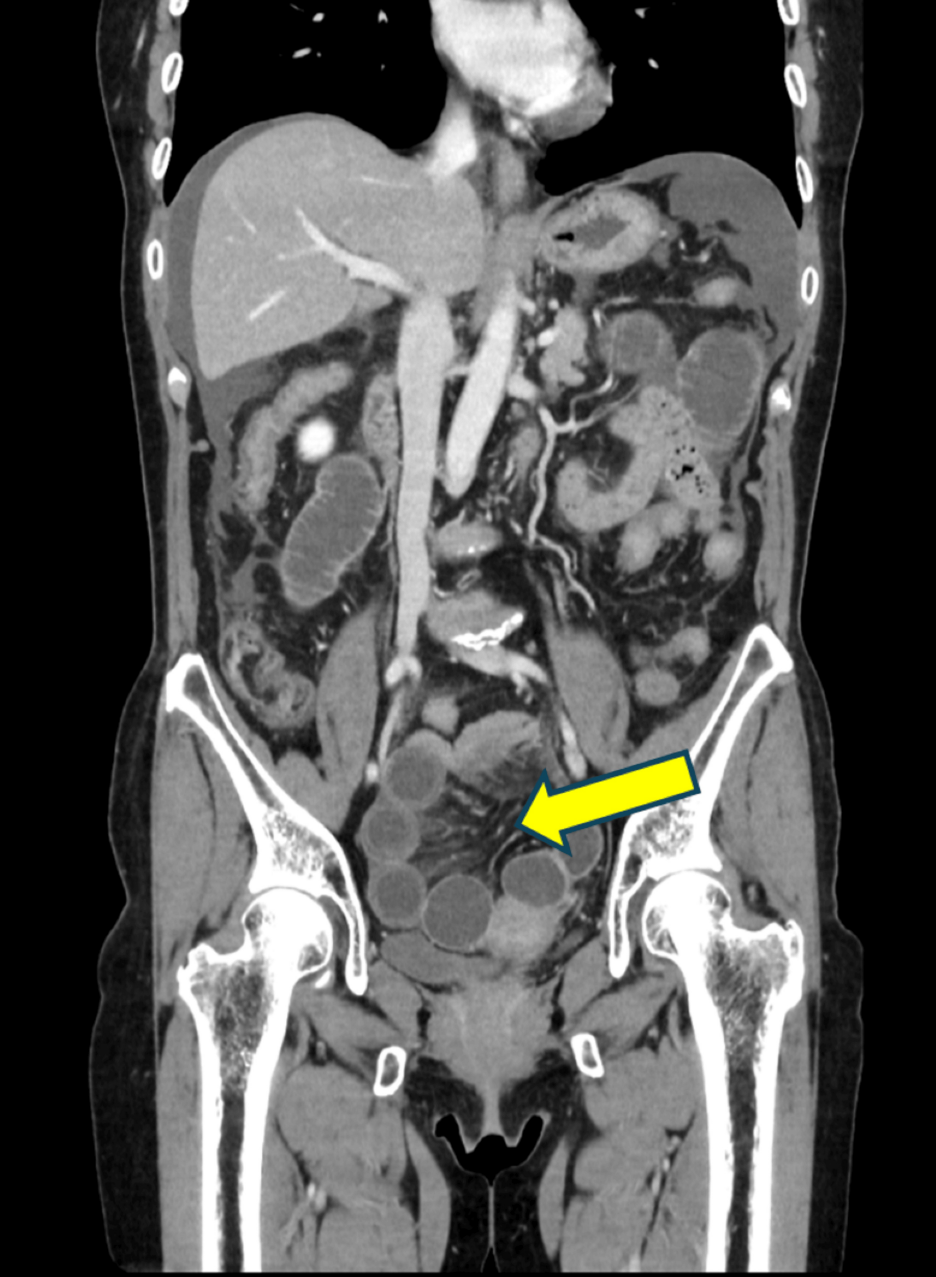
Contrast-enhanced abdominal CT revealed convergence of the mesentery near the uterus.

Abdominal CT revealed a small amount of ascites around the liver and pelvic floor, along with marked small bowel dilatation. A transition point was identified near the mesentery on the left side of the uterus, forming a closed loop. Contrast-enhanced CT demonstrated an ileal loop within the Douglas pouch, which displaced the uterus anteriorly and to the right. Two beak signs were observed on the left side of the uterus (Figure 2), and the mesentery converged near the uterus (Figure 3), indicating the transition point of the closed-loop obstruction.

Based on these findings, the patient was diagnosed with strangulated bowel obstruction due to a left-sided uterine broad ligament hernia, and emergency laparotomy was performed.

Surgical findings

**Figure 4:**
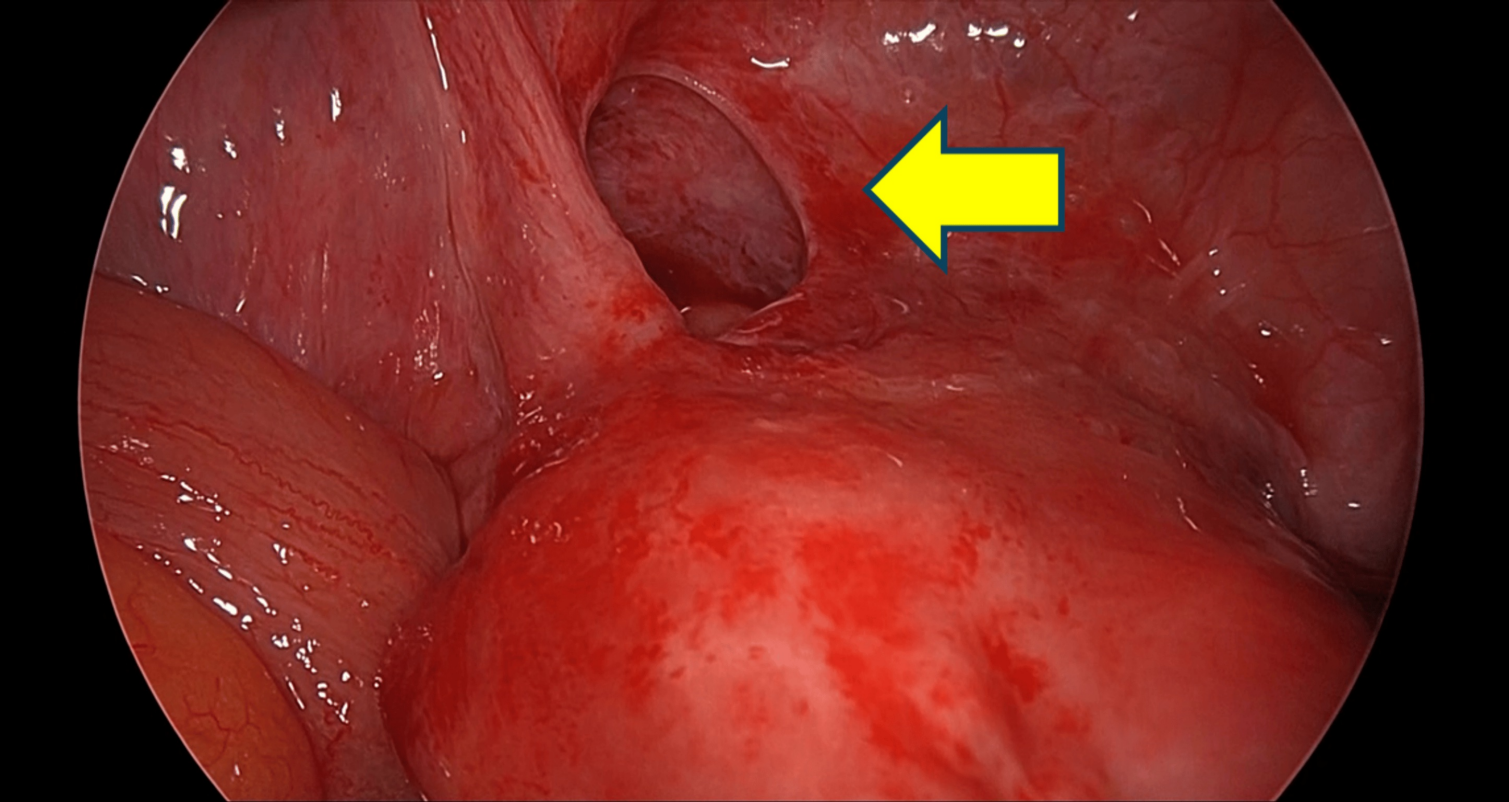
A defect was observed in the left broad ligament of the uterus.

Under general anesthesia, a lower midline laparotomy was performed, revealing dark reddish ascitic fluid. Exploration of the abdominal cavity identified an ileal segment located approximately 40 cm proximal to the terminal ileum, which was incarcerated within a defect in the left broad ligament of the uterus (Figure 4). The herniated small intestine was carefully reduced by gentle extraction from the hernial orifice. The strangulated bowel segment appeared brownish and showed reduced peristalsis. More than 24 hours had passed from symptom onset, and bowel necrosis was suspected. To assess blood flow, intraoperative indocyanine green (ICG) fluorescence imaging was used.

**Figure 5:**
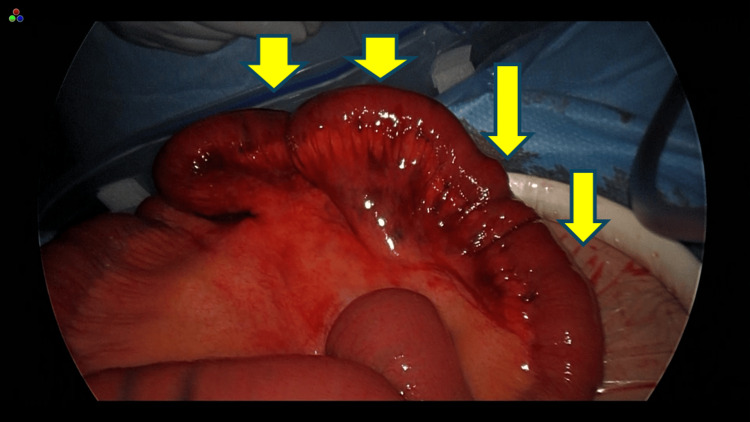
Under conventional light observation, the incarcerated bowel appeared brownish in color.

**Figure 6:**
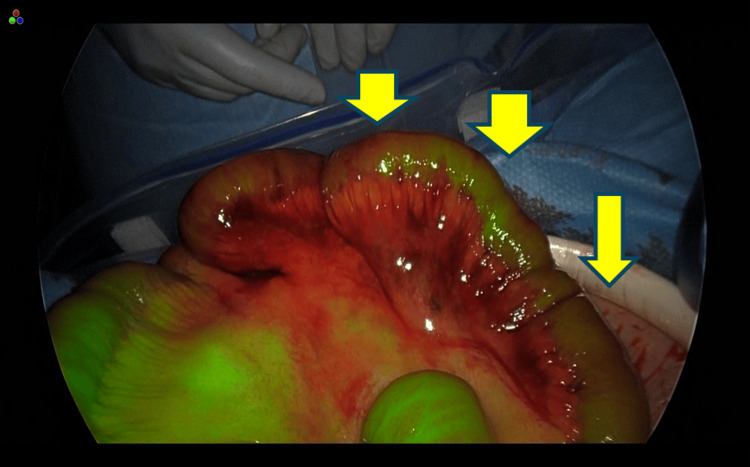
Under infrared light observation, uniform fluorescence was detected throughout the entire incarcerated bowel segment.

A total of 25 mg of ICG was dissolved in 10 mL of normal saline, and 5 mL of the solution was administered intravenously. Fluorescence imaging using a laparoscopic system was performed to evaluate perfusion. Homogeneous fluorescence was observed throughout the previously incarcerated bowel segment 20 seconds after intravenous injection of ICG, confirming adequate blood flow (Figures 5-6). Since perfusion was preserved, bowel resection was deemed unnecessary, and the intestine was preserved.

The hernial orifice, which measured approximately 2 cm in diameter, was closed using non-absorbable sutures. Another defect in the right broad ligament, measuring 4 cm in width, was also identified and closed with sutures. The total operation time was 2 hours and 6 minutes, and the estimated blood loss was 30 mL.

Postoperative course

On postoperative day (POD) 1, the nasogastric tube was removed, but due to paralytic ileus, it was reinserted on POD 3. After signs of improvement, the tube was removed again on POD 5, and oral intake of fluids was resumed. On POD 6, the patient was able to restart oral food intake. Her postoperative course remained uneventful, and she was discharged on POD 10. Subsequent outpatient follow-up revealed no complications, and she was deemed to have fully recovered.

## Discussion

Uterine broad ligament hernia is a rare condition in which the bowel or other abdominal organs become incarcerated within a defect or sac of the broad ligament.

This condition is classified into two types. The 'fenestra type' is characterized by defects in both the anterior and posterior peritoneal layers of the broad ligament, whereas the 'pouch type' involves a defect in only one of these layers. The fenestra type is more common, and the present case falls into this category. The etiology includes congenital anomalies, mechanical factors such as childbirth or surgery, tissue adhesions and distortions resulting from pelvic infections, and a reduction in the elasticity of the broad ligament due to aging [2].

Preoperative diagnosis is often difficult, but with advancements in imaging techniques, some cases can now be identified based on characteristic CT findings [3]. These findings include an incarcerated small bowel loop within the Douglas pouch, anterior or lateral displacement of the uterus due to the small bowel loop, and convergence of the mesentery near the uterus. A PubMed search using the keywords 'uterine broad ligament hernia' and 'preoperative diagnosis' identified 15 cases, including the present case. Among them, preoperative diagnosis was achieved in 9 cases (60%). Imaging findings showed that an incarcerated small bowel loop was present in all cases (100%), uterine displacement was observed in 11 cases (73%), and mesenteric convergence was seen in 4 cases (27%).

In our case, CT imaging revealed leftward and anterior displacement of the uterus, a caliber change suggesting an obstruction mechanism on the right side of the uterus, and stretching of the broad ligament, which enabled a preoperative diagnosis of broad ligament hernia. When these characteristic CT findings are observed, early suspicion of broad ligament hernia is crucial, as preoperative diagnosis allows for timely intervention.

Surgical treatment involves reducing the incarcerated bowel, resecting necrotic segments if necessary, and closing or widening the hernial defect. ICG fluorescence imaging is particularly useful for assessing bowel perfusion. In recent years, it has been widely used in gastrointestinal surgery to evaluate perfusion in reconstructed intestinal segments and to assess ischemic areas [4]. Boni L et al. reported that colorectal resection was performed in 107 patients undergoing laparoscopic colorectal surgery based on fluorescence intensity, with none of these patients developing clinical anastomotic leakage [5]. Nakashima K et al. reported that among 14 cases diagnosed with strangulated ileus, bowel resection was performed in four cases based on fluorescence intensity. Histopathological examination confirmed intestinal necrosis, and no intraperitoneal complications were observed [6].

In our case, surgery was performed more than 24 hours after symptom onset. Given the possibility of bowel preservation, intraoperative ICG fluorescence imaging was used to confirm sufficient intestinal perfusion, allowing for bowel preservation without resection. No postoperative complications occurred, highlighting the potential usefulness of ICG fluorescence imaging in assessing bowel viability and guiding surgical decision-making.

## Conclusions

Early preoperative diagnosis based on characteristic CT findings facilitated timely surgical intervention. Intraoperative ICG fluorescence imaging was essential for assessing bowel perfusion, enabling bowel preservation despite prolonged strangulation. This case underscores the significance of detailed preoperative imaging and the role of ICG fluorescence imaging in optimizing surgical decision-making.
